# Complex *PKD1* Genetics in Early-Onset Cystic Kidney Disease

**DOI:** 10.34067/KID.0000000000000110

**Published:** 2023-03-30

**Authors:** Matthew B. Lanktree

**Affiliations:** Departments of Medicine and Health Research Methodology, Evidence & Impact, Division of Nephrology, St. Joseph's Healthcare Hamilton & McMaster University, Hamilton, Ontario, Canada

**Keywords:** ADPKD, human genetics

In this issue of *Kidney360*, Gulati *et al.*^1^ present a case series of five severe pediatric-onset patients with cystic kidney disease resulting from compound heterozygous *PKD1* variants. In two of the patients, there is an affected parent with a classical *PKD1* frameshift variant, often referred to as a “protein-truncating mutation” in the autosomal dominant polycystic kidney disease (ADPKD) literature, with a missense variant coming from the other unaffected parent, while in the other three patients, the pediatric-onset disease is reported to be the result of compound heterozygous rare missense variants. Do these patients prove the observed *PKD1* missense variants partially reduce polycystin function (*i.e*., that they are hypomorphic)? Should *PKD1* be considered a cause of autosomal recessive polycystic kidney disease?

Identifying genetic modifiers of ADPKD has proven difficult. Gulati *et al.*^1^ do not systematically search for additional genetic contributors to the severe observed phenotypes, where rare predicted pathogenic variants in additional cytogenic genes (*GANAB*, *DNAJB11*, *HNF1B*, *IFT140*, *PKHD1*, *ALG8*, *ALG9*, *DZIP1L*, *COL4A3/4/5 etc.*) or other genes across the exome could contribute to the severe phenotype through digenic inheritance. However, even if a more thorough examination of the genetic background for further genetic modifiers or contributors was performed, assessing the additional variants' contribution to the phenotype would be difficult. Environmental modification of cystic kidney disease severity is less likely in these early-onset patients. Certainly, the bilineal inheritance of two rare *PKD1* variants seems the most likely explanation for the severe phenotypes, although the pathogenicity of the variants is unclear.

The first step when evaluating a patient with two identified rare variants in one gene is to determine their phase. Mendel's law of independent assortment arose from his observation that his pea plants were equally likely to produce smooth or wrinkled seeds regardless if the seeds were green or yellow, as the traits were coded by genes on different chromosomes. Linkage disequilibrium is the phenomenon whereby genetic variants that are located near each other on a chromosome are more likely to be passed down to offspring together than chance and hence are exceptions to Mendel's law. To a modern geneticist, two variants inherited on the same chromosome from one parent are known as “*in cis*,” while if they arose from each parent, they are on opposite chromosomes and “*in trans.*” Practically, determining the phase of two identified rare variants (*i.e.*, whether they are *in cis* or *in trans*) is difficult in a singleton patient and is best performed by examining the genotype of siblings or parents. If a parent has one, but not both variants, the proband is likely a compound heterozygote with the variants *in trans*, and both of the patient's copies of the gene carry a rare variant. By contrast, if one parent has both variants, they are in linkage disequilibrium *in cis*, it becomes difficult to disentangle the effects of the two variants, and the proband has one *wild type* copy of the gene and one copy of the gene carrying the two rare variants in linkage disequilibrium. In all five of the presented patients, the authors were able to demonstrate that the rare variants were likely *in trans*.

Next, the pathogenicity of the identified variants needs to be assessed. This is relatively straightforward in the case of a loss-of-function protein-truncating variant (*i.e*., nonsense or frameshift), but clinicians caring for patients with suspected genetic conditions are likely familiar with the challenges of assessing missense variants of uncertain significance or “VUS.” Individually rare variants are cumulatively common in the genome, and bioinformatic prediction algorithms and databases of variants identified in affected patients can lead to overestimates of the pathogenicity of variants.^2^ This overestimation has been documented across numerous phenotypes because the cumulative prevalence of these predicted pathogenic variants is greater than the clinically observed prevalence of the disease.^3,4^ Gulati *et al.*^1^ report the uncertain and conflicting interpretations of the pathogenicity of these observed “second hit” *trans PKD1* missense variants.

In addition, described by the authors, the currently prevailing hypothesis is that ADPKD severity is proportional to the quantity of polycystin-1-polycystin-2 complex that is successfully trafficked to the cilia of the renal tubular epithelial cells.^5^ Haploinsufficiency, resulting from a truncating variant, reduces the gene dosage by 50%. Acquired somatic mutations in the *wild type* copy of the gene could further reduce the polycystin complex dosage. Variants in genes affecting polycystin trafficking through the endoplasmic reticulum can also reduce the mature polycystin complex at the cilia.^5^ Homozygous loss-of-function *PKD1* variants have not been observed in humans and have long been presumed incompatible with life.^6^ How “*PKD1* gene dosage” is measured or predicted for a specific variant remains an area of research. Clinically, given the variable disease expressivity and incomplete penetrance of pathogenic variants, imaging and rate of eGFR decline remain the best means to risk stratify ADPKD severity.

The current case series is certainly in line with previous reports that homozygous missense *PKD1* variants can lead to severe early-onset cystic presentations.^7^ The Human Gene Mutation Database and ADPKD Mutation Database include 114 patients of compound heterozygotes and nine patients with homozygous variants, 14 of which were associated with severe *in utero* or pediatric-onset disease.^8^ Curiously, compound heterozygous missense *PKD1* variants that are predicted to be pathogenic have also been reported in eight unrelated patients with febrile seizures and epilepsy, although none had enlarged kidneys or kidney cysts, again demonstrating the challenges of variant interpretation, pathogenicity prediction, and incomplete penetrance.^9^

In sum, while the pathogenicity of hypomorphic variants remains difficult to pin down for individual patients, the current case series emphasizes that homozygous or compound heterozygous missense *PKD1* variants should be considered in patients with *in utero* or pediatric-onset severe cystic kidney disease, a phenocopy that can mimic autosomal recessive polycystic kidney disease.

## Disclosures

M. Lanktree reports the following: Research Funding: I have grant funding from the Canadian Institutes of Health Research, Hamilton Health Sciences, the Canadian Kidney Foundation, and Hamilton Academic Health Sciences Organization (HAHSO); Honoraria: Bayer, Otsuka, Reata, Sanofi; Advisory or Leadership Role: MBL received compensation for participating in advisory and consultancy boards with Bayer, Otsuka, Reata, Sanofi; and Speakers Bureau: Bayer, Otsuka, Reata, Sanofi.

## Funding

None.
